# Shaping the bodily self: exploring the influence of early attachment experiences on body ownership

**DOI:** 10.1007/s00426-026-02280-y

**Published:** 2026-03-26

**Authors:** Maria Pyasik, Lorenzo Pia, Carlotta Fossataro, Francesca Garbarini, Margherita Bruno, Mauro Adenzato, Rita B. Ardito

**Affiliations:** 1 Laboratory of Cognitive Neuroscience, Department of Languages and Literatures, Communication, Education and Society, University of Udine, Udine, Italy; 2 SAMBA Research Group, Department of Psychology, University of Turin, Turin, Italy; 3 Neuroscience Institute of Turin – NIT, Turin, Italy; 4 Psychology Department, MANIBUS LAB, University of Turin, Turin, Italy; 5 Human Science and Technologies, University of Turin, Turin, Italy; 6 Department of Psychology, University of Turin, Turin, Italy

**Keywords:** Body ownership, Rubber hand illusion, Attachment theory, Internal working models, Attachment styles

## Abstract

Body ownership is commonly studied using the rubber hand illusion (RHI), an experimental paradigm that induces a temporary feeling of owning a fake hand. Although the illusion demonstrates that multisensory integration is at the core of body ownership, factors like personality traits also modulate the experience and/or intensity of the illusion in a top-down manner. Here, we investigated the role of early attachment experiences as a factor that could influence body ownership. The RHI was administered at baseline and after a short-term activation of the attachment system using the Adult Attachment Projective (AAP). Proprioceptive drift, subjective ownership, and disownership were compared between the two conditions. Participants’ attachment style (*Free*, *Dismissing*, and *Entangled*), as determined by the Adult Attachment Interview (AAI), was considered a stable, between-subject (trait-like) index of adult attachment representations. The RHI effects were modulated by the attachment style, but not by the transient activation of the attachment system. Specifically, proprioceptive drift was larger in the synchronous compared to the asynchronous condition only for participants with the *Free* attachment style, whereas the subjective aspect of the RHI was not modulated by attachment style. These results suggest that individuals with *Free* attachment style are more sensitive to the multisensory integration underlying the RHI than individuals with *Dismissing* or *Entangled* attachment styles, which may reflect greater flexibility in visuo-proprioceptive recalibration under ambiguity. Thus, early attachment experiences may selectively influence the recalibration of lower-level body-related visual and proprioceptive information, but not the higher-level subjective attribution of body parts to oneself.

## Introduction

Body ownership, i.e., the feeling that one’s body, or a part of it, belongs to oneself, is a central component of self-awareness since humans experience the world and interact with it and others only through their bodies. Being ubiquitous, body ownership should necessarily influence multiple aspects of self-representation, and it is therefore crucial to understand its mechanisms.

One of the most employed research methods to investigate body ownership is the Rubber Hand Illusion (RHI), first described by Botvinick and Cohen (1998). The RHI is an experimental paradigm that is able to create a temporary illusory feeling of owning a fake hand, the so-called embodiment. During the RHI, a fake (mannequin or prosthesis) hand is placed congruent with the participant’s shoulder, parallel to the corresponding real hand, which is hidden from view. Synchronous, but not asynchronous, touches are then applied to the fake and real hands, creating the illusion of feeling the touches on the fake hand and perceiving the fake hand as part of one’s body. The illusion is usually quantified via subjective reports and a behavioral measure called proprioceptive drift. The former is assessed with ad hoc questionnaires concerning various aspects of embodiment, such as ownership of the fake hand, referral of touch to the fake hand, and disownership (loss of ownership) of one’s hand (Longo et al., 2008). The latter refers to a perceived shift in the position of one’s hand towards the fake hand following the synchronous visuotactile stimulation (Botvinick & Cohen, 1998). In this study, we use the RHI as an experimental probe of multisensory bodily self-processing, focusing on recalibration of self-localization and related body ownership experiences under induced sensory ambiguity.

Given the temporal and spatial constraints of the RHI described above, the key mechanism subserving the RHI is considered to be the multisensory integration (i.e., the process of integrating visual, tactile, and proprioceptive signals) with top-down effects of the pre-existing body model (Carruthers, 2013; Tsakiris, 2010; Tsakiris et al., 2010). It is worth noting, however, that recent evidence suggests that the strength of the illusion may also be affected by a number of factors related to personality: empathic concern (Asai et al., 2011; Durgin et al., 2007), psychosis proneness (Asai et al., 2011; Germine et al., 2013; Kállai et al., 2015; Thakkar et al., 2011), emotional intelligence (Perepelkina et al., 2017), personality traits (Burin et al., 2019), sensory suggestibility (Marotta et al., 2016), and affective disposition towards the body (Romeo et al., 2025). Within such a frame of reference, another personality-related factor that naturally may exert a significant top-down modulation on the multisensory integration processes is the one related to the early attachment experiences.

The attachment theory (Bowlby, 1969/1980) provides a framework to understand how early interactions with primary caregivers influence socio-emotional development and, ultimately, the mental representations of the self. The idea is that infants are neurobiologically predisposed to form emotional attachments with their caregivers in order to create a secure base for exploration and a safe haven in times of distress. The nature of these early relationships leads to enduring patterns of emotion regulation, expectations of others, and interpersonal behaviors. In adults, these early attachment experiences are assessed with the Adult Attachment Interview (AAI) (George et al., 1996) in which individuals have to reflect on their relationships with their attachment figures in childhood as well as the impact of these relationships on their development (see Methods for details). In the present study, adult attachment is treated as a relatively stable, between-subject (trait-like) factor operationalized via AAI classifications (*Free*, *Dismissing*, *Entangled*) (George et al., 1996; Main et al., 2003; Ravitz et al., 2010). During the AAI, the attachment-related discourse reveals the structure and organization of a person’s Internal Working Models (IWMs), namely the mental representations that guide expectations, emotions, and behaviors in close relationships (Bowlby, 1969/1980; Bretherton & Munholland, 2008). These IWMs are expressed in characteristic attachment styles, that is, relatively stable strategies for seeking proximity and regulating distress, and can be understood as cognitive–affective models that organize how people anticipate others’ responsiveness, experience themselves as worthy of care, and manage attachment-related arousal in close relationships (Cassidy, 1994; Mikulincer & Shaver, 2016). Because attachment regulation is inherently embodied, these models also shape how bodily states such as arousal, comfort, and threat are noticed and interpreted, contributing to bodily and self-representations. In turn, such stable differences in IWMs, as reflected in AAI classifications, provide a plausible mechanism through which adult attachment style could influence multisensory integration, by biasing how internal and external bodily cues are combined when constructing a coherent sense of one’s body (Fotopoulou & Tsakiris, 2017; Krahé et al., 2016; Oldroyd et al., 2019). From this perspective, IWMs may affect susceptibility to the RHI, particularly the recalibration of proprioceptive localization during synchronous multisensory stimulation. Thus, attachment theory, operationalized through the AAI and the concept of IWMs, provides a powerful lens for examining the enduring influence of early relationship experiences on psychological functioning and interpersonal dynamics in adulthood.

It is worth noting that converging findings suggest that the models mentioned above are also deeply rooted and grounded in early multisensory interactions with primary caregivers [e.g., (Fotopoulou & Tsakiris, 2017; Luo et al., 2024; Schore, 2022)]. In particular, early relational experiences, especially those based on embodied and affectively charged exchanges, appear to play a fundamental role in shaping the bodily self, a core level of self-awareness that integrates interoceptive, proprioceptive, and affective cues [e.g., (de Klerk et al., 2021; Gallagher, 2000; Montirosso & McGlone, 2020)]. Indeed, from birth, the infant is embedded in a relational matrix of rhythmic, contingent multisensory stimuli — including skin-to-skin contact, affective touch, vocal modulation, and synchronized movement — that occur within the caregiving dyad [e.g., (Ronga et al., 2021; Stern, 1985; Ulmer Yaniv et al., 2021; Wass et al., 2022)]. These embodied interactions not only serve to regulate the infant’s physiological state but also promote the development of a coherent and continuous bodily self that allows the infant to gradually differentiate between self and other and develop early forms of bodily agency and boundary awareness (Trevarthen & Aitken, 2001; Tronick, 2007). Additionally, such body-to-body relational experiences with the caregiver contribute to the development of a pre-reflective, sensorimotor sense of self, which serves as a foundation for later socio-emotional and cognitive development [e.g., (Meltzoff & Marshall, 2020)]. These experiences facilitate the integration of bottom-up bodily signals into higher-order representations and link attachment processes with embodied self-organization. Previous studies that investigated the interplay between the attachment processes and the bodily self-representation showed that differences in attachment styles were associated with the levels of interoceptive awareness (Oldroyd et al., 2019), perception of affective and social touch (Beltrán et al., 2020; Ekeberg, 2016; Krahé et al., 2018; Spitoni et al., 2020), and modulation of pain perception by affective touch (Krahé et al., 2016). Therefore, the early multisensory interactions are fundamental for the creation and maintenance of a robust and flexible bodily self. Nevertheless, no study to date has directly investigated the role of the attachment processes in body ownership. Although direct evidence linking attachment representations to multisensory integration mechanisms is still limited, converging findings indicate that attachment-related differences are expressed in the processing of bodily signals that are central inputs to embodiment, such as interoceptive awareness, affective and social touch perception, and the modulation of pain by affiliative touch [e.g., (Krahé et al., 2016; Oldroyd et al., 2019; Spitoni et al., 2020)].

Building on theoretical accounts that emphasize the social and developmental shaping of bodily self-experience, we propose that attachment may influence low-level sensory recalibration indirectly, by shaping momentary affective and regulatory states (e.g., arousal and threat/safety appraisal) and, in turn, how attention is allocated to internal (proprioceptive/interoceptive) versus external (visual/tactile) cues under bodily ambiguity (Fotopoulou & Tsakiris, 2017; Seth, 2013; Tsakiris, 2010). From this perspective, attachment-related differences are not expected to alter basic sensory encoding, but to modulate how bodily cues are combined when constructing self-location, which is what proprioceptive drift is thought to index in the rubber hand illusion.

Although attachment theory is inherently developmental, most evidence linking early relational experiences to bodily self-processing remains indirect, and stronger causal claims require longitudinal or developmental designs that track caregiving, attachment representations, and embodied self-processing over time (Fraley, 2002; Waters et al., 2000). This adult study, therefore, provides an initial test of whether individual differences in adult attachment representations are associated with variability in multisensory embodiment, while highlighting the need for future developmental work to examine when and how these links emerge.

For a comprehensive understanding of attachment dynamics, one must consider both their enduring patterns over time (i.e., attachment styles as assessed by the AAI) and their situational activation (i.e., short-term triggers by Adult Attachment Projective). Attachment phenomena have been described as ‘state-dependent traits’ (Ravitz et al., 2010), which emphasizes the dual nature of attachment: while there is a stable, trait-like consistency in individuals’ attitudes towards close relationships, these attitudes are not constantly expressed. Instead, they are typically activated by specific circumstances, especially when associated with danger, threat, or isolation. Therefore, to capture transient, state-dependent activations, we used the Adult Attachment Projective [AAP, (George, 2009; George & West, 2001, 2011)] to temporarily activate the attachment system immediately before the RHI paradigm. The AAP is a standardized, picture-based narrative task in which participants describe attachment-relevant scenes. It is designed to activate the attachment system and elicit attachment-related defensive and regulatory responses. It thus provides an index of momentary, state-like activation of attachment processes, complementing the trait-like assessment obtained from the AAI (see Methods for more details).

Based on the theoretical background described above, the present study aims to test whether adult attachment representations, as determined by AAI classifications, are associated with individual differences in RHI responses, particularly the recalibration of self-localization (visuo-proprioceptive integration). Specifically, we examine whether individual differences in adult attachment style are associated with variations in RHI indices, with a specific focus on proprioceptive drift as a behavioral marker of self-localization recalibration, and on subjective ratings as a complementary measure of conscious body ownership experience. To our knowledge, no previous studies have directly examined the relationship between adults’ attachment style and the RHI susceptibility. Given this gap in the literature, the present study adopts an exploratory approach to investigate the potential long-term influence of early relational experiences on multisensory bodily self-representations.

During the study, the RHI was conducted under two conditions: at baseline, i.e., as a standalone procedure, and immediately after the short-term activation of the attachment system by administering the AAP. We measured the strength of the RHI with proprioceptive drift and subjective ratings of body ownership and disownership, and evaluated whether: (1) RHI susceptibility and strength were related to participants’ attachment style as determined by the administration of the AAI in a separate session prior to the RHI sessions, and (2) RHI effects would be modulated by the short-term activation of the attachment system by the administration of the AAP right before the administration of the RHI. Here, “short-term activation of the attachment system” refers to a temporary increase in the salience of attachment-related concerns elicited by attachment-relevant cues. The AAP is widely used as an attachment-activating stimulus because its picture-based narratives reliably evoke themes of vulnerability, separation, and care-seeking, thereby engaging attachment-related appraisal and regulation. Empirical work indicates that AAP exposure can recruit neural systems involved in processing attachment-relevant scenes and is accompanied by changes in stress- and affiliation-related physiology (Buchheim et al., 2016; Krause et al., 2016). We assumed that this temporary activation could shift participants’ momentary affective and neurocognitive state (e.g., arousal and threat/safety appraisal), which may bias multisensory integration by altering attentional weighting and the relative reliance on internal (proprioceptive/interoceptive) versus external (visual/tactile) bodily cues under ambiguity. Converging evidence also suggests that attachment activation via AAP can induce short-term changes in resting-state EEG functional connectivity, consistent with a transient shift in neurocognitive state following attachment-related stimulation (Carbone et al. 2025a, b).

Building on the Internal Working Model (IWM) framework described above, we hypothesized that adult attachment (indexed by AAI classifications) would modulate the multisensory integration processes underlying the RHI. Specifically, we predicted greater RHI susceptibility in individuals with a Free attachment classification compared to insecure classifications (Dismissing and Entangled), with effects expected to be most evident in proprioceptive drift – a behavioral index of proprioceptive recalibration – during synchronous rather than asynchronous stimulation. Given the exploratory nature of the study and the lack of specific prior evidence linking AAI insecure subtypes to differential RHI profiles, we did not expect a priori distinct patterns between Dismissing and Entangled classifications; instead, we treated both as insecure groups expected to show reduced RHI susceptibility compared to the Free group and examined any differences between insecure subtypes exploratorily. Finally, we explored whether transient activation of the attachment system via the AAP would modulate RHI responses, potentially amplifying attachment-related differences in RHI susceptibility across conditions.

## Methods

### Participants

35 healthy right-handed volunteers (24 women, 11 men; mean age ± SD = 21.08 ± 1.26) with no history of neurological or psychiatric disorders gave written informed consent to participate in the study. All participants had normal or corrected-to-normal vision. Participants were recruited among the students at the University of Turin. The study was approved by the Bioethics Committee of the University of Turin (protocol number 122571) and was conducted in accordance with the ethical standards of the 2013 Declaration of Helsinki.

Two participants were excluded from the analyses because their attachment style could not be classified using the AAI (see Results section for details). Therefore, the final sample included 33 participants. The sample size was not estimated a priori due to the study’s exploratory nature. A sensitivity power analysis performed in G*Power (Faul et al., 2007) confirmed that this sample size (*n* = 33), with alpha level = 0.05 and power = 0.80 in a 3 × 2 × 2 mixed-effects ANOVA, was sufficient to detect a medium effect size (*f* = 0.24).

### Study design

The study consisted of three sessions. In the first session, which took place approximately 3 to 4 months before the other two sessions, participants were interviewed using the AAI, which allowed categorizing them in three groups according to their attachment style (free, F, entangled, E, and dismissing, Ds, see below for details). In the second and third sessions, the RHI measures were collected using identical RHI procedures. One of these two sessions (Baseline session) consisted of the RHI only, while the other included the administration of the AAP immediately prior to the RHI (Post-AAP session). The Baseline session was conducted to measure baseline participants’ susceptibility to the RHI. The Post-AAP session was used to investigate the possible modulation of the RHI strength by the activation of the attachment system after the AAP. The two RHI sessions were conducted one week apart, and their order was counterbalanced across participants.

The interval between the AAI session and the two RHI sessions was primarily due to practical reasons, such as scheduling constraints and the time required for interview transcription and coding, and was not intended as an experimental manipulation. Importantly, AAI classifications are generally considered relatively stable, trait-like indices of adult attachment representations. Therefore, we assumed that attachment classification would remain stable over the period separating sessions, as supported by evidence of test–retest stability and predictive validity of the AAI across months or years in non-clinical samples (Bakermans-Kranenburg & Van IJzendoorn, 2009; Fraley, 2002; Ravitz et al., 2010).

**Fig. 1:**
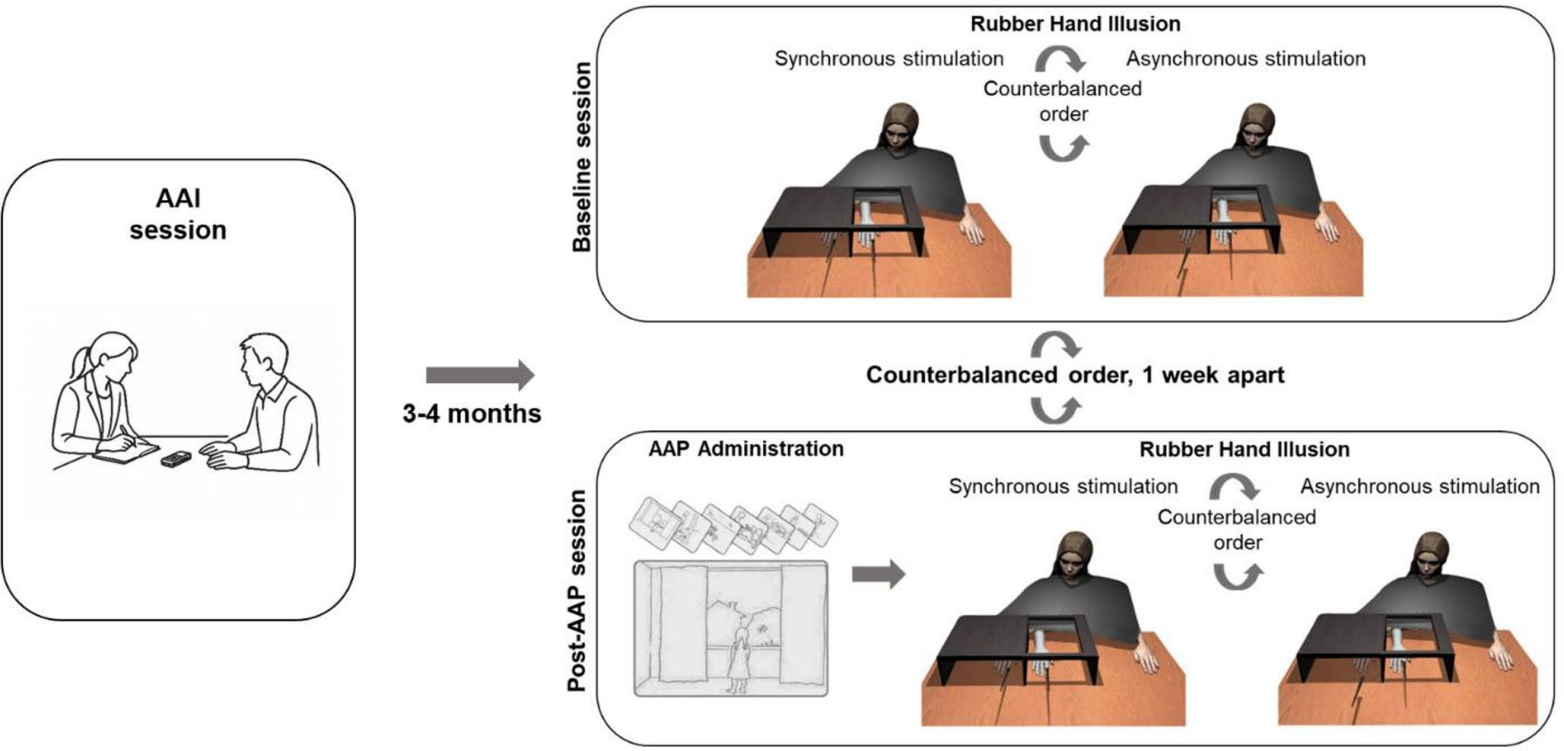
Experimental setup and procedure. The RHI procedure (synchronous and asynchronous conditions) was performed in two sessions, without other tasks preceding it (Baseline), or following the AAP administration (Post-AAP)

The study therefore had a 3 × 2 × 2 design with Attachment style (F, E, Ds) as a between-subject factor, and RHI Condition (Synchronous, Asynchronous) and Session (Baseline, Post-AAP) as within-subject factors. The following sections describe the procedures in detail and illustrated in Fig. 1.

### Setup and procedures

#### Adult attachment interview (AAI)

The aim of the AAI is to assess an adult’s mental representations of early attachment experiences, i.e., the structure of a person’s IWM. Previous rigorous studies, including meta-analyses and psychometric studies, have shown that the AAI has solid psychometric properties — such as stability and predictive and discriminant validity — in both clinical and non-clinical samples (Bakermans-Kranenburg et al., 2025; Bakermans-Kranenburg & Van IJzendoorn, 1993, 2009; Ravitz et al., 2010; Sagi et al., 1994).

The AAI was conducted during the first session of the study. Upon arrival, participants were led to a comfortable and quiet room where a trained clinical psychologist conducted the interview, which lasted an average of 1.5 h. The interview involved a series of questions in a predetermined order that gradually led the person to recall childhood memories (from the earliest memories to the age of 12) related to their parents, including relationship and emotional memories, moments of vulnerability, such as separations or rejections, and possible experiences of loss and abuse. In addition, a series of questions asked the person to evaluate early attachment experiences and reflect on how these experiences might influence current and future relationships, such as parenting.

The whole interview was recorded, then transcribed, and finally coded using the Adult Attachment Scoring and Classification System (Main et al., 2003) into the three-way distribution of attachment, i.e., *Free* (F), *Dismissing* (Ds), and *Entangled* (E) attachment styles. The coding parameters are based on the degree of coherence between episodic and semantic memory and in the narrative as a whole. The degree of collaboration with the interviewer and the ability to metacognitively monitor when recalling childhood memories are also part of the parameters.

The three categories have the following main characteristics: *Free* individuals provide coherent, balanced, and reflective accounts of positive or negative childhood experiences; they demonstrate the ability to value attachment relationships and integrate their meaning. *Dismissing* individuals tend to minimize or idealize attachment experiences and often show emotional distance and a lack of memory; their narratives may be brief, contradictory, or overly positive without providing evidence. *Entangled* individuals are characterized by preoccupied, confused, or emotionally charged narratives, often involving anger or passivity about early relationships. Finally, according to the Adult Attachment Scoring and Classification System (Main et al., 2003), if the narrative shows no strategy and the state of mind shifts between Dismissing and Entangled strategies, the category of *Cannot Classify* is applied.

#### Adult attachment projective (AAP) picture system

The AAP (George, 2009; George & West, 2001, 2011) is a projective-narrative instrument that measures core attachment characteristics through a semi-structured interview. The AAP is frequently used in clinical and research settings due to its predictive validity, stability, and time efficiency (Ravitz et al., 2010). The interview takes place in a quiet room and lasts an average of 30 min. A series of black-and-white drawings, containing only enough detail to identify the selected event, is presented in a fixed order by a trained clinical psychologist. The sequence of drawings is designed to gradually activate the attachment system as they depict scenes reminiscent of the primal elements of attachment, such as loneliness, separation, death, illness, and abuse (i.e., attachment activators) (George & West, 2011). For each picture, the respondent is asked to describe what is happening in the picture, what led to the story event, what the characters are thinking or feeling, and what will happen next. To create a narrative, the respondent must retrieve their mental representations of attachment. Furthermore, some AAP pictures depict adult/adult and adult/child interactions, prompting the participant to access the mental availability of internalized attachment figures at different stages of life. The entire interview is recorded.

The present study used the AAP to activate the neurobiological attachment system before the RHI paradigm. It was administered before the RHI in one of the experimental sessions (the second or the third, depending on the order of sessions assigned to each participant, which was labelled the “Post-AAP” session), see Fig. 1, right bottom panel.

#### Rubber hand illusion

The RHI procedure followed the classical horizontal setup (Botvinick & Cohen, 1998). The participant was seated in front of a table with a wooden box (60 × 40 × 20 cm) placed on it. The box was divided into two halves with a vertical panel; one was closed on the top, and the other was open for view. A flat wooden panel of the same size (60 × 40 cm) was used to cover the top of the box. During the preparation phase, the top of the box was covered from view with the wooden panel, and the participant’s right hand and forearm were placed, palm down, on the table in the closed half of the box, which was further from the body midline than the open half. A prosthetic right hand and forearm, gender-matched with the participant, was placed in the open half of the box and aligned with the participant’s right shoulder. The space between the box and the participant’s neck was covered with a black barber sheet to conceal the arms from view and facilitate the impression that the fake hand was connected with the participant’s arm and shoulder. The RHI setup in the two conditions is presented in Fig. 1, right panels.

The participant was, firstly, instructed to keep their right hand relaxed and still for the entire task duration. Then, a tailor ruler was placed on top of the closed box, and the participant was asked to indicate the perceived position of their right index finger by naming the number on the ruler that corresponded to it. The experimenter took note of the difference between the actual and the perceived position of the index finger (in cm; positive values indicated a perceived mislocalization towards the fake hand, while the negative values indicated a perceived mislocalization away from the fake hand). This was referred to as *pre-stimulation proprioceptive judgment*, and the measure was repeated ten times. Between the trials, the ruler was moved horizontally several times and repositioned for the subsequent trial to avoid number repetitions. After the pre-stimulation proprioceptive judgments, the ruler was taken away, and the top of the box was opened, thus making the fake hand visible. The participant was instructed to maintain the gaze on the fake hand and remain still while the experimenter applied touches with paintbrushes on the index finger of the fake hand and the index finger of the corresponding real hand. The touches to the two hands were either synchronous (i.e., delivered simultaneously at approximately 1 Hz) or asynchronous (i.e., delivered in opposite phases at the same frequency as the synchronous stimulation), depending on the experimental condition (Synchronous and Asynchronous as experimental and control conditions, respectively). This *visuotactile stimulation phase* lasted 2 min and was followed by the *post-stimulation proprioceptive judgments*. In detail, after the stimulation, the top of the box was covered again, and the ruler was placed on the box; the instructions and the procedure were identical to the pre-stimulation measures. This part of the procedure was followed by the *embodiment questionnaire* that evaluated the subjective experience of the RHI. The questionnaire comprised 9 statements – 3 statements describing the illusory experience related to body ownership, 3 statements related to the experience of disownership of one’s real hand, and 3 control statements that evaluated the general compliance with the task instructions (see Table 1). The statements were based on previous RHI studies (Botvinick & Cohen, 1998; Longo et al., 2008) and administered in randomized order. Participant was instructed to rate their agreement with each statement on a 7-point Likert scale ranging from − 3 (complete disagreement) to + 3 (complete agreement).

The two experimental conditions (Synchronous and Asynchronous) were administered in counterbalanced order. Between the conditions, the participant was asked to take their right hand out of the box and move it to avoid the residual effects of the illusion. The entire RHI procedure lasted approximately 20 min.

**Table 1:** Embodiment questionnaire; the agreement with each statement was rated on a −3 to + 3 Likert scale, where − 3 indicated complete disagreement and + 3 complete agreement

Illusion (Ownership)	Q1. It felt as if I was feeling the touch of the paintbrush in the location where I saw the rubber hand touched.
	Q2. It felt as if the touch I felt was caused by the paintbrush touching the rubber hand.
	Q3. I felt as if the rubber hand was my hand
Control	Q4. It felt as if my hand was drifting towards the rubber hand.
	Q5. It seemed as if the touch I was feeling came from somewhere between my own hand and the rubber hand.
	Q6. It felt as if my hand was turning ‘rubbery’.
Disownership	Q7. It felt as if I was no longer able to move my hand.
	Q8. It felt as if I could no longer tell where my hand was located.
	Q9. It felt as if my hand had disappeared.

### Statistical analysis

The *Proprioceptive drift* was calculated as the difference between the post-stimulation and the pre-stimulation proprioceptive judgments (Tsakiris & Haggard, 2005) and averaged across trials. A positive value indicated the perceived shift in the location of one’s hand towards the fake hand after stimulation. The proprioceptive drift values violated the criteria for normality of distribution in the Shapiro-Wilk test. Hence, they were analyzed with a 3 × 2 × 2 mixed-effects ANOVA on aligned-rank transformed data [ART-ANOVA; (Wobbrock et al., 2011)], with Attachment style (F, E, Ds) as a between-subject factor, and RHI Condition (Synchronous, Asynchronous) and Session (Baseline, Post-AAP) as within-subject factors.

The *embodiment questionnaire ratings* were averaged between Q1-Q3 to obtain the *ownership* score, Q4-Q6 to obtain the control items score, and Q7-Q9 to obtain the *disownership* score. The ownership and control scores were entered into a 3 × 2 × 2 × 2 ART-ANOVA with Attachment style (F, E, Ds) as a between-subjects factor, and RHI Condition (Synchronous, Asynchronous), Session (Baseline, Post-AAP), and Question type (Ownership, Control) as within-subject factors. The disownership scores were entered into a 3 × 2 × 2 ART-ANOVA for Attachment style x Condition x Session.

When significant interactions were present, post-hoc paired comparisons were performed using the Tukey HSD to adjust for multiple comparisons.

## Results

### Adult attachment interview

In accordance with the AAI coding system, 15 participants (42.9%) were classified as having *Free* attachment style; 11 participants (31.43%) were classified as having *Entangled* attachment style, and 7 participants (20%) had *Dismissing* attachment style. These percentages are consistent with those recently determined by Bakermans-Kranenburg et al. (2025), p. 204 in a meta-analysis of more than 2000 AAIs administered to European individuals using the three-way distribution method. The interviews of two participants revealed no strategy in the narrative, and therefore, these participants were assigned to the *Cannot Classify* category and subsequently excluded from the analyses.

### Rubber hand illusion: proprioceptive drift

A 3 × 2 × 2 ART-ANOVA showed a significant main effect of *Condition* [F(1,30) = 13.77, *p* < 0.001, η_p_^2^ = 0.31; mean ± SD Synchronous = 1.00 ± 1.88, Asynchronous = −0.16 ± 1.77] and a significant interaction of *Condition x Attachment style* [F(2,30) = 4.66, *p* = 0.017, η_p_^2^ = 0.24; mean ± SD Synchronous Ds = 0.51 ± 1.80, Synchronous E = 0.86 ± 1.85, Synchronous F = 1.34 ± 1.94; Asynchronous Ds = 0.77 ± 1.06, Asynchronous E = 0.00 ± 1.44, Asynchronous F = −0.70 ± 2.06]. Post-hoc paired comparisons revealed that the proprioceptive drift was significantly higher in the Synchronous condition compared to Asynchronous only in the F group (p_adj_ < 0.001) but not in the E and Ds groups (p_adj_ = 0.96 in Ds and p_adj_ = 0.47 in E).

There were no significant main effects of *Session* [F(1,30) = 0.03, *p* = 0.87, η_p_^2^ < 0.001; mean ± SD Baseline = 0.49 ± 1.96, Post-AAP = 0.36 ± 1.87], or *Attachment style* [F(2,30) = 0.20, *p* = 0.82, η_p_^2^ = 0.01; mean ± SD Ds = 0.64 ± 1.45, E = 0.43 ± 1.69, F = 0.32 ± 2.24], or other interactions: *Condition x Time* [F(1,30) = 1.60, *p* = 0.22, η_p_^2^ = 0.05];

*Time x Attachment style* [F(2,30) = 2.30, *p* = 0.15, η_p_^2^ = 0.01]; *Condition x Time x Attachment style* [F(2,30) = 1.93, *p* = 0.16, η_p_^2^ = 0.11].

**Fig. 2:**
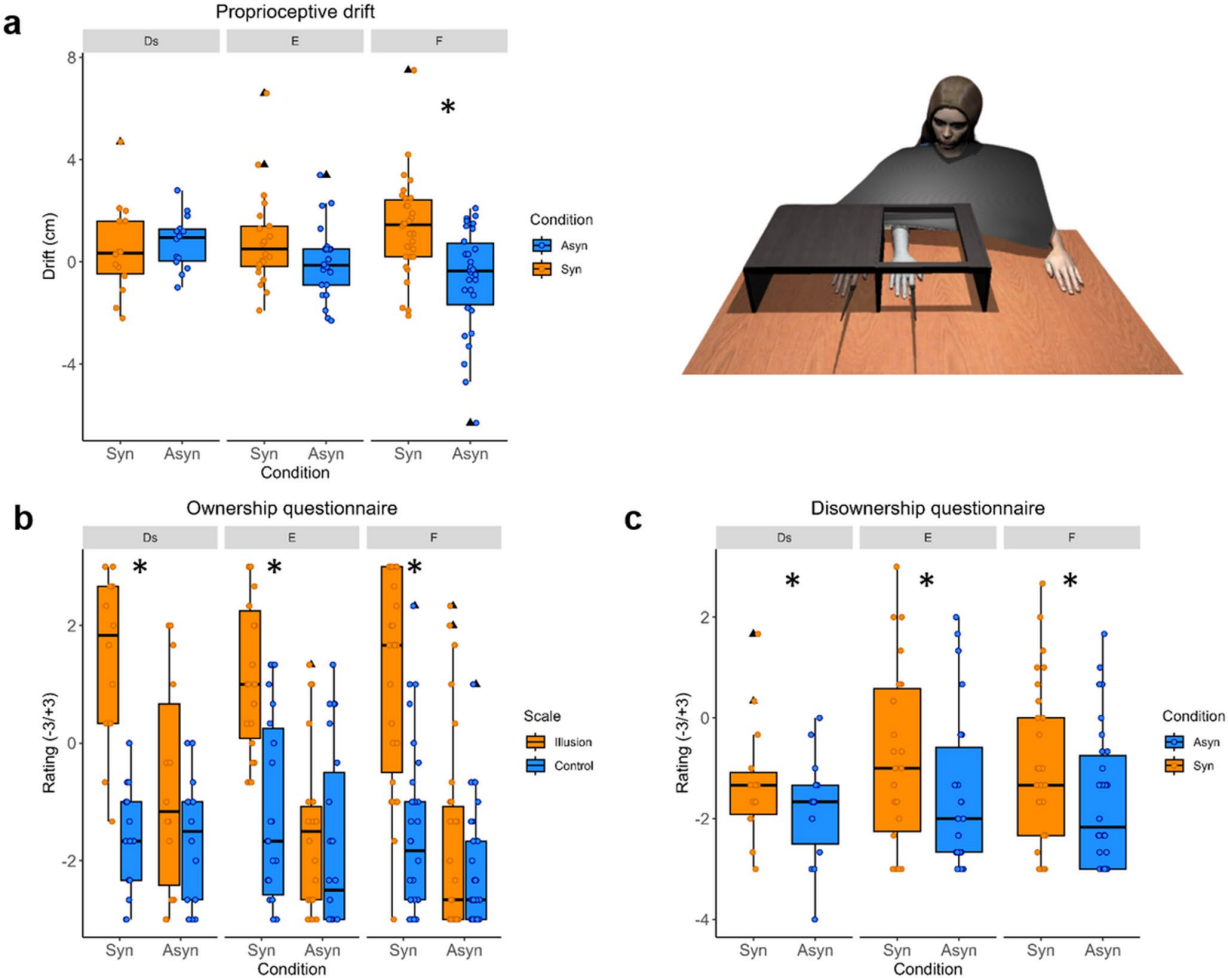
RHI results in the three attachment styles: **a.** Proprioceptive drift (cm) in two experimental conditions (Synchronous and Asynchronous) in the three groups according to the attachment style; **b**. Ratings in the Illusion (Ownership) and Control statements of the embodiment questionnaire (−3 to + 3) in the three groups according to the attachment style; **c.** Ratings in the Disownership statements of the embodiment questionnaire (−3 to + 3) in the three groups according to the attachment style. In all panels, the hinges of the box represent the first and the third quartile, with the line in the middle of the box representing the median, and the bottom and top whiskers representing the smallest and the largest value no further than 1.5*IQR from the lower and the upper hinges, respectively. The black triangles represent the outliers; the smaller dots represent individual values. Syn = Synchronous, Asyn = Asynchronous, Ds = Dismissing, E = Entangled, F = Free. * = significant differences

To summarize, the proprioceptive drift was significantly larger after synchronous stimulation compared to asynchronous stimulation only in the group with a Free attachment style. This effect was not modulated by the administration of the AAP, i.e., it was comparable between baseline and post-AAP sessions. The results are shown in Fig. 2a.

### Rubber hand illusion: embodiment questionnaire

Regarding the *ownership* and the *control* statements of the questionnaire, a 3 × 2 × 2 × 2 ART-ANOVA showed a significant main effect of *Condition* [F(1,30) = 71.61, *p* < 0.001, η_p_^2^ = 0.70; mean ± SD Synchronous = −0.14 ± 1.99, Asynchronous = −1.70 ± 1.46], with significantly higher ratings in Synchronous than in Asynchronous condition, and a significant main effect of *Question type* [F(1,30) = 58.14, *p* < 0.001, η_p_^2^ = 0.66; mean ± SD Illusion = −0.13 ± 2.07, Control = −1.70 ± 1.34], with significantly higher ratings in Illusion (ownership) questions compared to Control ones. Furthermore, there was a significant interaction of *Condition x Question type* [F(1,30) = 57.22, *p* < 0.001, η_p_^2^ = 0.66; mean ± SD Synchronous Illusion = −1.20 ± 1.55, Synchronous Control = −1.46 ± 1.64, Asynchronous Illusion = −1.47 ± 1.41, Asynchronous Control = −1.93 ± 1.24]. Post-hoc paired comparisons indicated that the ratings were significantly higher in the Illusion statements compared to the Control ones only in the Synchronous condition (p_adj_ < 0.001) but not in the Asynchronous (p_adj_ = 0.11); in addition, the ratings in Illusion statements were significantly higher in Synchronous condition compared to the Asynchronous (p_adj_ < 0.001). In addition, a significant interaction of *Session x Attachment style* was present [F(2,30) = 4.90, *p* = 0.01, η_p_^2^ = 0.25; mean ± SD Baseline Ds = −0.51 ± 1.60, Baseline E = −1.09 ± 1.83, Baseline F = −1.04 ± 1.92, Post-AAP Ds = −0.80 ± 2.06, Post-AAP E = −0.57 ± 1.85, Post-AAP F = −1.17 ± 2.06]. However, post-hoc paired comparisons revealed no significant effects.

In summary, the ratings in the illusion (ownership) statements were significantly higher than the respective control statements only in the condition with the synchronous stimulation; they were also significantly higher in the synchronous condition compared to the asynchronous one. There were no significant effects of either attachment style, or the experimental session (Post-AAP). The results are presented in Fig. 2b.

As regards the *disownership* ratings, according to a 3 × 2 × 2 ART-ANOVA, there was a significant effect of *Condition* [F(1,30) = 7.59, *p* = 0.01, η_p_^2^ < 0.001; mean ± SD Synchronous = −1.01 ± 1.60, Asynchronous = −1.63 ± 1.40], with significantly higher ratings in Synchronous than in Asynchronous condition. No other main effects or interactions resulted in being significant (see Fig. 2c).

## Discussion

The present study investigated whether trait-like attachment styles in adults were related to the susceptibility and strength of RHI and whether the effects of RHI could be modulated by short-term activation of the attachment system, i.e., whether these effects are state-dependent. Our results showed that proprioceptive drift, an indicator of self-localization and referral of touch, was significantly larger in the Synchronous than in the Asynchronous condition, and only in participants classified as having a *Free* attachment style according to the AAI. Attachment style was not associated with subjective embodiment scores, including ownership and disownership. Both ownership and disownership ratings were significantly higher in the synchronous, compared to the asynchronous, condition, and significantly higher ratings in the illusion statements describing ownership compared to the control statements. Overall, this suggests that the fake hand was subjectively embodied only in the synchronous condition, regardless of the attachment style. Furthermore, activation of the attachment system with the AAP did not significantly modulate the RHI effects across groups.

The results of the RHI followed the classic RHI pattern (Botvinick & Cohen, 1998), that is the larger proprioceptive drift after the synchronous stimulation compared to the asynchronous (only in one group of participants, as described above), as well as higher ratings in the illusion vs. control statements of the embodiment questionnaire only in the synchronous condition. Moreover, subjective disownership of one’s own hand corresponding to the embodiment fake hand followed the same pattern as ownership of the fake hand, i.e., the disownership ratings were significantly higher in the synchronous condition. However, in both conditions, the ratings were negative, indicating a general tendency to disagree with the explicit disownership statements, which closely mirrors previous findings showing negative or only weakly positive disownership ratings [e.g., (Kannape et al., 2019; Longo et al., 2008; Pyasik et al., 2020)]. Together, these converging results support the notion that disownership is a genuine, albeit phenomenologically weaker, component of embodiment compared to ownership of the fake hand, possibly because participants’ attention is primarily directed toward the fake hand during the RHI (Lane et al., 2017). However, it is worth noting that the subjective experience of embodiment assessed with questionnaires might be influenced by non-specific factors such as general susceptibility to experimentally induced illusions, demand characteristics, suggestibility, or responsiveness to the experimental context [for discussions, see (Ehrsson et al., 2022; Lush et al., 2020, 2021; Slater & Ehrsson, 2022)]. Therefore, while the present findings are strongly grounded, in and consistent with, the existing literature, they should be interpreted with appropriate caution and not as a selective or pure index of alterations in body ownership per se.

The fact that the drift, but not subjective feelings of body ownership, was related to the a *Free* attachment style, is consistent with the hypothesis that this style, which is typically associated with coherent and flexible IWMs (Main et al., 2003), supports a more flexible self-representation that allows for greater plasticity in the integration of congruent multisensory information [e.g., (Ferraro & Taylor, 2021; Oldroyd et al., 2019)]. Previous research demonstrates that securely attached individuals (i.e., *Free* individuals) exhibit better emotion regulation, higher interoceptive accuracy, and more effective integration of self-relevant information [e.g., (Martín Quintana et al., 2023; Mikulincer & Shaver, 2019; Oldroyd et al., 2019)], all factors that may facilitate a more plastic and integrated bodily self. More broadly, secure (Free) attachment has been linked to greater regulatory capacity and context-sensitive flexibility in processing self-relevant information, which could translate into more adaptive and potentially more flexible functional coupling within multisensory networks during embodiment [e.g., (Mikulincer & Shaver, 2016; Vrticka & Vuilleumier, 2012)]. Conversely, individuals with *Dismissing* or *Entangled* attachment styles tend to hyperactivate or deactivate attachment-related affective cues (Mikulincer & Shaver, 2016, 2019), which could contribute to less flexible sensory processing and more rigid or dysregulated bodily self-representations. For example, attachment-related avoidance (characteristic of *Dismissing* individuals) has been associated with lower sensitivity to interoceptive and affective body cues, while anxious attachment (characteristic of *Entangled* individuals) correlates with hypervigilance and fragmented self-processing (Oldroyd et al., 2019; Vrticka & Vuilleumier, 2012). Such patterns may impair top-down modulation of multisensory integration, resulting in a weaker or less coherent embodiment response, as was observed in our sample, in which *Dismissing* and *Entangled* participants showed no significant differences in proprioceptive drift.

The observed relation between the attachment style and the proprioceptive drift, but not the subjective feeling of ownership, might be explained by the partially different mechanisms subserving the two RHI outcomes (Pyasik et al., 2018; Riemer et al., 2015; Rohde et al., 2011). Indeed, the proprioceptive drift could be primarily based on the integration of visual and proprioceptive information from visuotactile stimulation (Litwin, 2020). It reflects self-localization (Carruthers, 2013; Dempsey-Jones & Kritikos, 2014) and may be dissociated from feeling of ownership (Holle et al., 2011; Rohde et al., 2011). In turn, the subjective experience of ownership requires not only synchronous multisensory stimulation, but also the constraints related to the pre-existing body model, i.e., the anatomically plausible posture and position of the fake hand. Therefore, it relies more on visual and somatosensory information (Litwin, 2020), which is influenced by higher-level constructs, such as the body model (Gallagher, 1986; Pyasik et al., 2020).

This dissociation between proprioceptive drift (self-localization) and body ownership in RHI is further supported by neuroimaging data showing that a brain network involving parietal (posterior parietal cortex and inferior parietal sulcus) and premotor (particularly, ventral premotor cortex) areas is activated during RHI [see (Castro et al., 2023) for a review]. Importantly, an fMRI study (Guterstam et al., 2015) directly investigated neural activation related to body ownership and self-localization as two distinct processes and showed that self-localization was related to activation of posterior cingulate, retrosplenial, and intraparietal cortices, and the hippocampus, while body ownership was supported by activation of premotor-intraparietal areas. Furthermore, these two groups of brain areas were functionally connected. This connection was mediated by the posterior cingulate cortex, indicating its crucial role in integrating self-localization and body ownership. This is consistent with the behavioral data and the theoretical considerations described above on the different underlying mechanisms of proprioceptive drift and body ownership within the general phenomenon of embodiment in RHI.

Rather than attempting to localize attachment effects to a single node within the RHI network, we interpret our findings at the level of functional components of embodiment. Specifically, the attachment-related modulation observed here – limited to proprioceptive drift – is consistent with a preferential influence on self-localization and visuo-proprioceptive recalibration, which have been linked to posterior midline and parietal contributions in previous neuroimaging studies (Guterstam et al., 2015), whereas explicit ownership ratings may depend more on premotor–parietal mechanisms supporting higher-level ownership attribution. Accordingly, our results are compatible with the possibility that adult attachment differences modulate self-localization-related processes more than ownership-related processes; however, this mechanistic interpretation remains tentative and should be tested directly in future studies combining attachment measures, the RHI, and neuroimaging. In this framework, attachment-related differences may plausibly bias multisensory weighting within the broader embodiment network, particularly the posterior midline and parietal contributions to self-localization, thereby affecting visuo-proprioceptive recalibration as indexed by proprioceptive drift.

To summarize, body ownership tends to depend on stable higher-order body representations, whereas proprioceptive drift reflects a recalibration of low-level visual and proprioceptive perception. Our results suggest that early attachment-related experiences selectively influence this lower-level recalibration rather than the subjective attribution of body parts to the self.

The prior activation of the attachment system by the AAP did not increase or decrease the strength of the illusion in any group. This might suggest that the modulation exerted by the attachment representations on the bodily self is not state-dependent, but rather trait-related and anchored in the person’s stable cognitive-affective structures. However, it could also be that the RHI paradigm is not sensitive enough to capture the transient changes caused by the activation of the attachment system, as the AAP-related modifications have been previously observed in other body-related paradigms, e.g., defensive behaviors (Fossataro et al., 2023).

There are several limitations to consider. First, the study’s exploratory nature and the limited previous literature on this topic limit the generalizability of the results. Future studies should aim to replicate these findings in larger and more diverse samples. Another limitation is related to the use of the AAP for the activation of the attachment system. While such activation has been shown in the literature (Buchheim et al., 2016; Krause et al., 2016), in the present study it was not measured directly (for example, through concurrent physiological indices or brief state ratings of arousal or affect following the AAP). Therefore, we cannot exclude that the lack of significant differences in the RHI effects between the baseline and post-AAP sessions might be related to the insufficient effects of the AAP. Furthermore, the present study allowed exploring the association between attachment style and body ownership, but not a causal relation between the two processes. Longitudinal or developmental studies could clarify how early multisensory interactions between caregivers and infants shape the bodily self over time, thus providing a possibility to establish a causal link. In addition, the integration of physiological or neuroimaging data could shed light on whether different neural circuits support the possible modulation of self-localization by attachment.

Despite these limitations, to our knowledge, this is the first study to examine attachment style in relation to susceptibility to the RHI and provides new evidence that early attachment experiences have a lasting impact on multisensory body processing. Future research should further investigate the complex interplay between attachment style, sensory suggestibility, and body ownership and extend these findings to clinical populations. In particular, disturbances of embodiment and body ownership have been documented in conditions characterized by interpersonal trauma and attachment-related difficulties (e.g., post-traumatic stress disorder and borderline personality disorder), making it important for future work to test whether attachment representations contribute to vulnerability for body ownership disturbances and whether attachment-focused interventions modulate embodiment-related processes [e.g., (Neustadter et al., 2019; Rabellino et al., 2018)]. From a broader perspective, these findings suggest that attachment theory may provide a fruitful framework for understanding interindividual variability in embodied self-awareness.

## Data Availability

Raw data of the AAI (audio recordings of the interviews and their transcripts) cannot be publicly shared in full to protect participants’ confidentiality. De-identified summary data relevant to the study analysis and findings can be made available upon reasonable request to the corresponding author (L.P.), in accordance with institutional ethical guidelines and data protection regulations.
